# An algorithm to compare two‐dimensional footwear outsole images using maximum cliques and speeded‐up robust feature

**DOI:** 10.1002/sam.11449

**Published:** 2020-02-21

**Authors:** Soyoung Park, Alicia Carriquiry

**Affiliations:** ^1^ Department of Statistics Iowa State University Ames Iowa

**Keywords:** forensic science, image comparison, machine learning, maximum clique, SURF

## Abstract

Footwear examiners are tasked with comparing an outsole impression (*Q*) left at a crime scene with an impression (*K*) from a database or from the suspect's shoe. We propose a method for comparing two shoe outsole impressions that relies on robust features (speeded‐up robust feature; SURF) on each impression and aligns them using a maximum clique (MC). After alignment, an algorithm we denote MC‐COMP is used to extract additional features that are then combined into a univariate similarity score using a random forest (RF). We use a database of shoe outsole impressions that includes images from two models of athletic shoes that were purchased new and then worn by study participants for about 6 months. The shoes share class characteristics such as outsole pattern and size, and thus the comparison is challenging. We find that the RF implemented on SURF outperforms other methods recently proposed in the literature in terms of classification precision. In more realistic scenarios where crime scene impressions may be degraded and smudged, the algorithm we propose—denoted MC‐COMP‐SURF—shows the best classification performance by detecting unique features better than other methods. The algorithm can be implemented with the R‐package shoeprintr.

**Abbreviations**
BART: Bayesian additive regression trees
CSAFE: Center for Statistics and Applications in Forensic Evidence
MC: maximum clique
MC‐COMP: maximum clique comparison
NRC: National Research Council
RAC: randomly acquired characteristic
RF: random forest
ROC: receiver operating characteristic
SURF: speeded‐up robust feature

## 1 INTRODUCTION

Forensic practice in the United States has been under intense scrutiny for several years. The development of DNA analysis as a forensic tool in the 1990s 13 provided the means to revisit old criminal cases and identify individuals who had been wrongfully convicted because of faulty forensic analyses. In addition, highly publicized blunders including the false accusation of Brandon Mayfield as the perpetrator in the 2004 Madrid train bombing also put in question the reliability of forensic techniques such as latent print analysis, forensic bite‐mark analysis, and other *pattern disciplines*. Pattern disciplines include fingerprint and shoeprint examination, ballistics, and other forensic disciplines where the evidence can be represented in the form of a two or three dimensional image. In the early 2000s, the National Research Council (NRC) published reports discussing the scientific validity and reliability of bullet lead analysis 15, lie‐detecting 14, eye witness testimony 18, and ballistics 16. But, it was the 2009 report by the NRC 17 that finally increased awareness of the lack of science underpinning many of the pattern disciplines. The report noted that even widely accepted techniques such as fingerprint comparison had not been tested under controlled conditions, and that discipline‐wide error rates were unknown. The panel made several recommendations, among them that the broader scientific community be recruited to work on forensic problems to put them on a more solid scientific and probabilistic framework. A few years later, the President's Council of Advisors on Science and Technology (PCAST) revisited the subject, and in a 2016 report 24 concluded that little progress had been made since 2009, and that there was still need for objective, well tested a validated methods to evaluate evidence. A recent paper by Bell et al. 4 pointed out that after almost a decade since the release of the 2009 NRC report, many forensic disciplines have yet to be validated. The paper by Bell and her co‐authors is a call to scientists in a wide range of areas to get involved in forensic science research.

In this article, we focus on footwear examination, one of the pattern disciplines discussed in both the 2009 NRC report 17 and the PCAST report 24. Both reports noted that the comparison of footwear impressions is largely subjective and relies heavily on the examiner's experience. Furthermore, PCAST expressed concerns about the potential lack of reliability and accuracy among footwear examiners and pointed out that when addressing questions of source we must consider the question of *probative value* of footwear evidence. Probative value refers to the *rarity* of the observed similarity; two items of evidence have high probative value if a strong degree of similarity is indicative of same source.

There is a need to develop objective, accurate and repeatable methods to compare footwear impressions. Despite the fact that footwear impressions are more commonly found in crime scenes than fingerprints 29, footwear evidence is rarely introduced as evidence in criminal proceedings. In fact, it is often the case that footwear evidence is not even lifted from the crime scene. This is so because crime scene investigators lack the tools and the knowledge to do so correctly 5 and footwear examiners lack accurate, reliable and validated methods to quantify the similarity between two outsole (the bottom of the shoe) impressions. Consequently, examiners are limited in the type and strength of conclusions they can make.

The state of the art in footwear examination is to compare two or more outsole images visually and then use a 7‐point scale to subjectively determine the degree of similarity between them. The guidelines for implementing the 7‐point scale were published in a report entitled *Range of conclusions standard for footwear and tire impression examinations* in 2013 by the Scientific Working Group for Shoeprint and Tire Tread Evidence, which also includes suggestions for the wording to use when reporting conclusions.

The fact that footwear examination relies on a subjective assessment of the similarity between outsole impressions is problematic for various reasons. First, there is no universal agreement of what constitutes the degree of similarity associated with each point in the scale. Second, there is no mechanism to estimate error rates for the discipline as a whole and for individual examiners.

Unless the crime scene print is significantly degraded, it is relatively simple to exclude a suspect's shoe when it does not share class characteristics (such as size of the shoe, make, and model) with the questioned shoe impression at the scene. However, when two impressions share class characteristics, the only identifying marks would be those that arise from wear and tear. If the two impressions are found to be *similar enough*, then the next question is whether the observed degree of similarity is probative: could we observe the same degree of similarity if the two impressions had been produced by different shoes?

In the last decade, there have been several published papers 2, 11, 26, 27, 29, 31 proposing automated or semi‐automated algorithms to compare shoe outsole impressions. Yet, there has been no systematic evaluation of the performance of those methods on a database of two‐dimensional (2D) shoe impressions of outsoles with the same class characteristics. In addition, databases of images of outsoles obtained from well‐designed trials controlling for brand, model, size, and degree of wear are scarce, and this has impeded progress by the research community.

The main goal of this work is to introduce a statistical learning algorithm that can reliably determine whether a questioned outsole impression may have been made by a specific shoe. The algorithm produces a *score* that takes a value between 0 and 1, where higher scores correspond to higher degree of similarity between the impressions that are being compared. To develop and test the algorithm, we used 2D images of outsole impressions collected by the Center for Statistics and Applications in Forensic Evidence (CSAFE; http://www.forensicstats.org). The database can be accessed freely via the resources link in the center's webpage. The algorithm is not meant to replace the human examiner, but to provide an objective tool that the examiner can use to shore up visual examination.

The use of learning algorithms in forensic science applications is still experimental, but gaining popularity. In the past decade or so, learning algorithms in combination with more traditional statistical methods have been proposed for use in firearms examination 7, trace evidence 21, questioned documents 9, and footwear analysis 2, 11, 27, 31, just to name a few. In all cases, algorithms have been proposed as complementary to current forensic practice, and as a means to quantify the degree of similarity between two items as well as the probative value of the similarity. In the remainder, we describe the data and the algorithm, implement the algorithm on high quality and on degraded images and offer some conclusions.

## 2 DATA

**Figure 1:**
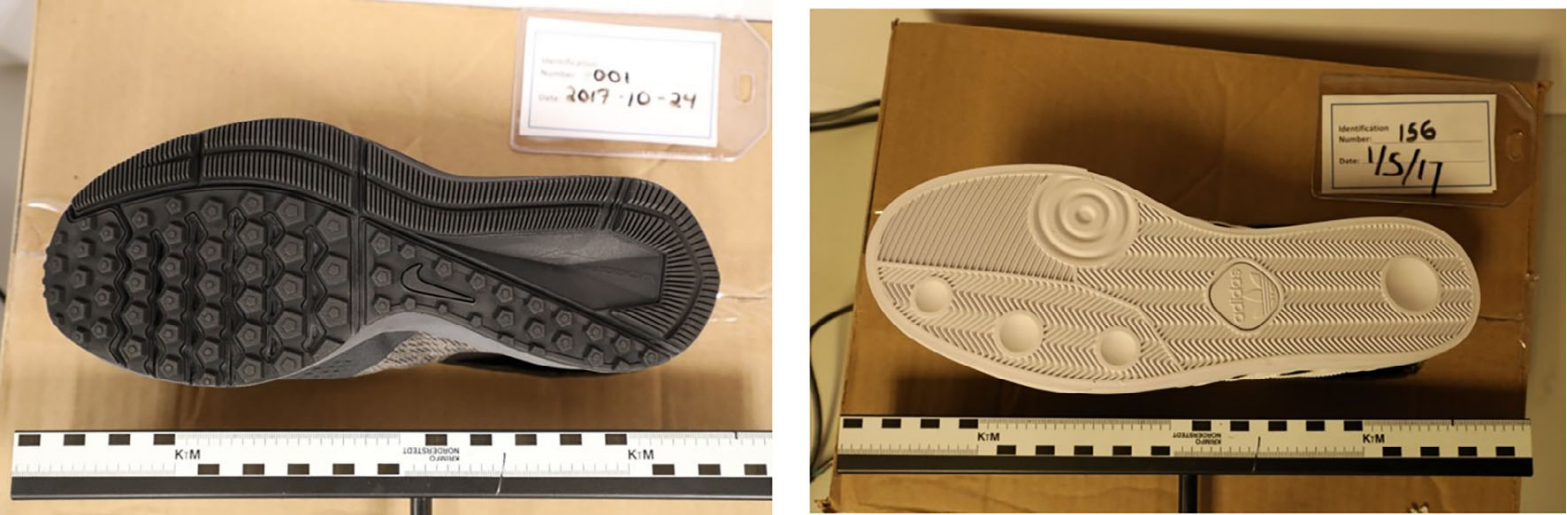
The outsole photos of Nike Winflow 4 and Adidas Seeley

Researchers in CSAFE obtained 2D images of shoe impressions for research purposes. One hundred and sixty new pairs of shoes of brand Nike Winflow 4, sizes 8.5 and 10.5 or Adidas Seeley skateboard, sizes 8 and 10 were purchased. Figure 1 shows the outsole pattern of study shoes. Volunteers were given a pair of brand‐new shoes equipped with a step counter, and were asked to walk at least 10 000 steps per week in the shoes. Participants brought the shoes back to the lab every 8 weeks over a 6‐month period so that outsoles could be scanned, photographed and printed. During each of these occasions, both the left and the right shoe were scanned four times. The resulting database includes over 30 000 images obtained using various devices in a longitudinal study design. Importantly, each image has a known source (or shoe).

One of the measurement instruments used in the study was a 2D EverOS scanner (https://www.shopevident.com/). The EverOS scanner obtains a 2D image of the outsole of the shoe by detecting the weight distribution on the outsole as the wearer steps on the scanner. The resolution of the image is 300 dpi and the image also includes a ruler to measure the size of the impression. For good quality images, participants carefully step on the scanner putting weight from the heel to the toe of the shoe, attempting to capture the entire shoe outsole.

In this paper, we use a subset of the images obtained during the last collection period, when shoes had been worn for about 6 months by study participants. The subset includes images of the left and the right shoe from 60 pairs of Nike Winflow 4 shoes size 8.5 (38 pairs), 10.5 (22 pairs) and from 21 pairs of Adidas Seeley skateboard shoes size 8 (11 pairs) and size 10 (10 pairs).

All images underwent some preprocessing: we removed the ruler areas in the boundary of the images and down‐sampled all images at a 20% rate. We used Matlab to implement these steps and also for detecting the SURF (speeded‐up robust features) 3. We revisit this step in the next section.

**Figure 2:**
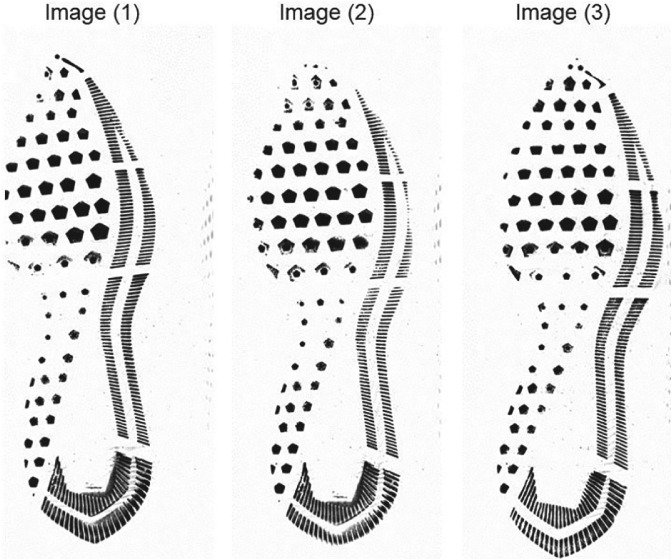
Three impressions of left Nike Winflow 4 shoes size 10.5, worn for about 6 months. Image 1: impression #1 from the left shoe of the pair with ID 144 scanned on May 2, 2018. Image 2: impression #3 from the left shoe of the same pair. Image 3: impression #1 from the left shoe of pair with ID 105 scanned on May 9, 2018. Image 1 and Image 2 are mates and Image 1 (or Image 2) and Image 3 are nonmates

Figure 2 shows three example impressions of the left shoe in pairs of Nike Winflow 4 size 10.5 shoes. Images 1 and 2 are were obtained from replicated impressions of the left shoe worn by participant #144. Image 3 is an impression from the left shoe worn by participant #105. Since the two pairs of shoes used to draw Figure 2 are of the same brand and model and are used for a similar amount of time, these three impressions look very similar. By the naked eye alone, it is challenging to see the differences in the impressions that are due to differences in wear and tear.

## 3 ALGORITHM TO COMPARE IMPRESSIONS

We propose an algorithm to quantify the similarity between pairs of images of footwear impressions. The goal is to build a classifier that can accurately predict whether two impressions have a common source, and produce a quantitative assessment of the strength of the similarity between the impressions.

The method we propose relies on three critical steps to construct a similarity score; we have attempted to optimize each of the steps to improve the predictive performance of the algorithm. In the first step we define an *outsole signature*, or a subset of the image pixels on which we will focus. The second step is to align two signatures and the last step is to measure the similarity between them.

### 3.1 The signature of an outsole

Rather than carrying out a pixel‐wise comparison of two images, it is typically more efficient to select *points of interest* from the outsole image. There are different ways of doing so; for example, chapter 3 in 20 extracted all edge pixels in footwear outsole images using a Prewitt operator 25. Other points of interest might include corner pixels, blobs, or scale‐invariant feature transforms (SIFT, 19), (KAZE 1), ORB (oriented FAST and rotated BRISK, 28). Desirable attributes of points of interest include reliability (the same points in the same positions are identified repeatedly), distinctiveness (features are uniquely captured), and robustness to noise and background effects. A comparison of the performance of several methods can be found in 30.

In this work, we rely on the strongest 500 SURFs 3, because the algorithm is invariant to changes in scale and rotation of images and is computationally efficient. Footwear outsole images like ours that have been down‐sampled at a 20% rate can have about 1000 SURFs. We opted for using 500 of the SURFs as a trade‐off between computational efficiency and classification performance of the algorithm.

### 3.2 Alignment

A method called maximum clique comparison (MC‐COMP) was introduced by Park (20, section 3.4.1) and discussed in Park and Carriquiry 23. MC‐COMP plays an important role in the alignment of images obtained from two shoes—a questioned and a known or reference shoe—which we label *Q* and *K*.

Suppose that we have extracted *n*_*Q*_ strong SURFs from the questioned outsole and transform them into (*x*, *y*) coordinate values. The coordinate values are anchored by coordinates (0, 0) in the lower left corner of the image. For the questioned impression (*Q*), the matrix of coordinate values corresponding to the SURFs is denoted by $S_{n_{Q},Q}$ as in expression (1). We define a similar matrix $S_{n_{K},K}$ (see expression (1)) to represent the coordinates of the SURFs in shoe *K*.

(1) $$S_{n_{Q},Q}=\left(\begin{array}{ccc} x_{1} & \ldots & x_{n_{Q}}\\ y_{1} & \ldots & y_{n_{Q}} \end{array}\right).$$

(2) $$S_{n_{K},K}=\left(\begin{array}{ccc} x_{1} & \ldots & x_{n_{K}}\\ y_{1} & \ldots & y_{n_{K}} \end{array}\right).$$

**Figure 3:**
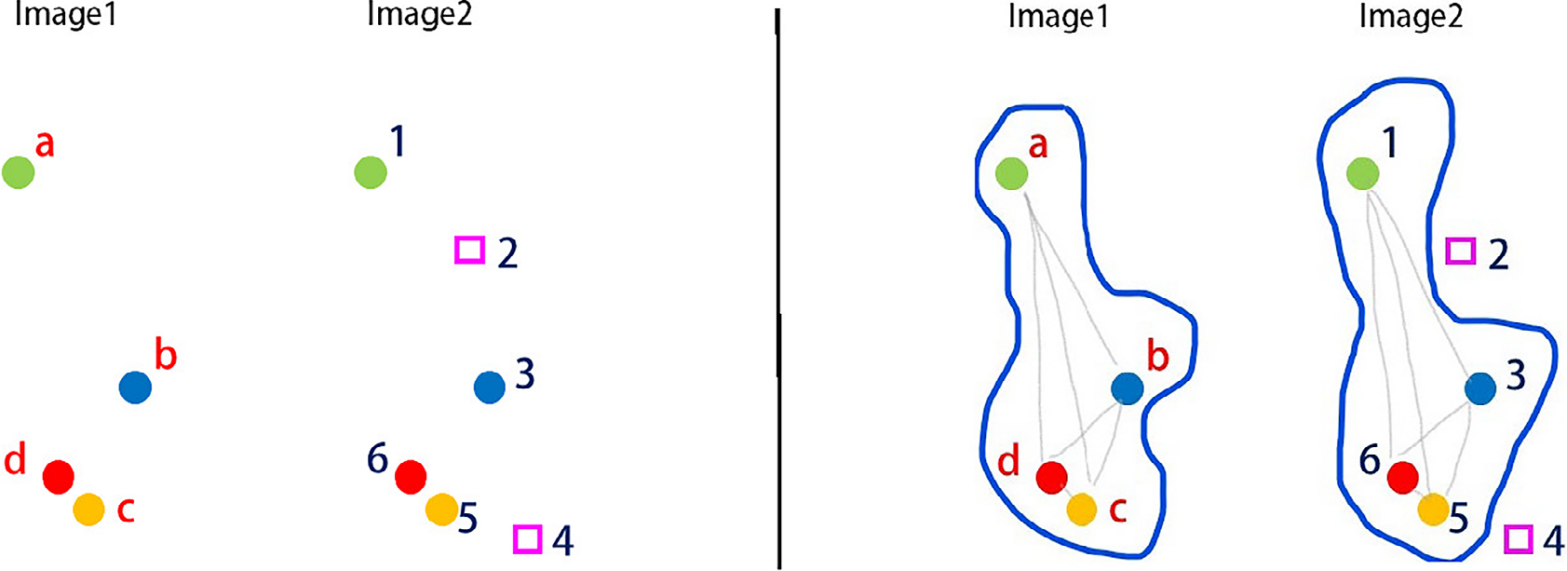
Left panel: Two images with 4 and 6 points each. Right panel: maximum clique of size 4

We rely on the concept of maximum clique (MC) to find subgroups of points in $S_{n_{Q},Q}$ and $S_{n_{K},K}$ with the same geometric arrangement. To identify a MC, the algorithm calculates all possible pairwise distances between points in each of $S_{n_{Q},Q}$ and $S_{n_{K},K}$ and then finds the subsets of points in each image that share a geometric arrangement. There are many such subsets; the one with the largest membership is called the MC. Figure 3 illustrates this idea. The two images on the left panel of the figure have four and six points, respectively. There are $\left(\begin{array}{c} 4\\ 2 \end{array}\right)$ pairwise distances in Image 1 and $\left(\begin{array}{c} 6\\ 2 \end{array}\right)$ in Image 2. Since the pairwise distances among all points in Image 1 are similar to those among points #1, #3, #5, and #6 in Image 2, the MC in this case has size four, and is shown on the right panel of the figure.

Finding the MC is a computationally demanding task; there are $\left(\begin{array}{c} 500\\ 2 \end{array}\right)=124,750$ possible pairwise distances between the 500 values in *S*_500,*Q*_. To speed up calculations, we select a subset of 100 points from $S_{n_{Q},Q}$. The subsampling process is the following. Image *Q* is divided into 100 equally sized bins and a single point is randomly selected within each bin. In that way, we construct a set of no more than 100 random points evenly distributed in the plane of *Q*. The set of sampled points in $S_{n_{Q},Q}$ is denoted $S_{n_{Q_{r}},Q_{r}}$. The algorithm proceeds as before, but now with a reduced set of points in *Q*: find the maximum set of corresponding points in $S_{n_{Q_{r}},Q_{r}}$ and $S_{n_{K},K}$, by comparing $\left(\begin{array}{c} n_{Q_{r}}\\ 2 \end{array}\right)$ pairwise distances in $S_{n_{Q_{r}},Q_{r}}$ and $\left(\begin{array}{c} n_{K}\\ 2 \end{array}\right)$ pairwise distances in $S_{n_{K},K}$.

The set of points in the MC between $S_{n_{Q_{r}},Q_{r}}$ and $S_{n_{K},K}$ is denoted *M*_*Q*,*K*_ and is shown in (3):

(3) $$\begin{array}{l} M_{Q,K}={\left\{\left(p_{1,Q},p_{1,K}\right),\ldots ,\left(p_{m,Q},p_{m,K}\right)\right\}}^{T}\\ p_{j,Q}=\left(x_{j,Q},y_{j,Q}\right),\;j=1,\ldots ,m\\ p_{j,K}=\left(x_{j,K},y_{j,K}\right),\;j=1,\ldots ,m. \end{array}$$

In (3), *M*_*Q*,*K*_ has size *m*. A point *p*_1,*Q*_ and a point *p*_1,*K*_ are in correspondence if the Euclidean distance between *p*_1,*Q*_ and *p*_2,*Q*_ is the same as the distance between *p*_1,*K*_ and *p*_2,*K*_. In a clique of size *m*, pairwise distances between *m* points in *Q* are the same as the pairwise distances between *m* points in *K*. Each point *p*_*i*,*Q*_ has coordinates *x*_*i*,*Q*_, *y*_*i*,*Q*_ for *i* = 1, …, *m*.

The MC obtained from $S_{n_{Q_{r}},Q_{r}}$ and $S_{n_{K},K}$ is an effective tool to align the two images by overlaying $S_{n_{Q},Q}$ and $S_{n_{K},K}$. Based on the *m* points in *M*_*Q*,*K*_, the algorithm calculates the rotation angle (*θ*) and a translation matrix (*T*_*A*_) to overlay $S_{n_{Q},Q}$ on to the plane of $S_{n_{K},K}$. We use the estimated *θ* and *T*_*A*_ to transform the entire set of points $S_{n_{Q},Q}$ so that they can be overlaid with the set of points $S_{n_{K},K}$:

$$\begin{array}{l} S_{n_{Q},Q}^{K}=\sum_{A}\times S_{n_{Q},Q}+T_{A}\\ =\left(\begin{array}{cc} \cos\;\theta & -\sin\;\theta \\ \sin\;\theta & \cos\;\theta \end{array}\right)\times S_{n_{Q},Q}+\left(\begin{array}{ccc} T_{x} & \ldots & T_{x}\\ T_{y} & \ldots & T_{y} \end{array}\right). \end{array}$$

### 3.3 Learning algorithm

Once the two sets of SURFs are aligned as described above, we can define several features to measure the similarity between the SURFs in *Q* and *K*. Here we focus on three different similarity features: (a) clique size, (b) % overlap, and (c) median distance (median Euclidean distance between overlapping points).

The *clique size* is defined as the number of SURFs in the MC, *m*. The feature that quantifies the degree of overlap between the two aligned images is calculated as follows. After alignment of $S_{n_{Q},Q}^{K}$ and $S_{n_{K},K}$, for each transformed point in $S_{n_{Q},Q}^{K}$, the algorithm finds the closest point in $S_{n_{K},K}$. If the distance between these two closest point (one is a transformed SURF in *Q* and the other is a SURF in *K*) is less than two units, then we say that the two points overlap (OP). The number of OP is denoted *n*_*OP*_ and by construction must be less than *n*_*Q*_ and *n*_*K*_. The threshold of two units is arbitrary, but appears to work well. The similarity feature *% overlap* is defined as the proportion of points in *Q* and *K* that overlap. The last similarity feature is the *median* (*Euclidean*) *distance* among OP points.

None of the summary features can individually determine the source of an image with an acceptable degree of accuracy. Consequently, an alternative is to construct a scoring metric that combines all the features into a single score that has good discriminating ability. Ideally, the resulting score is a numeric value with a bounded range, where high values of the score are associated with high (or low) degree of similarity between the images in a pair. Here, we combine the three similarity features using a random forest (RF; 6). Park and Carriquiry 21 used a random forest to combine the differences in 18 chemical compositions for a pair of glass fragments into a univariate score to decide whether fragments originated from the same pane of glass. A random forest outputs an empirical class probability for the binary response “mates” and “nonmates.” A pair of images is mated when the images were made by the same outsole. Nonmates are images made by different outsoles. We use the empirical probability associated with the “mated” class as our final similarity score.

The RF is a supervised learning algorithm, meaning that the algorithm *learns* on a training data set with known labels. Its classification performance is then *evaluated* on a different test set. Here, to construct the training set, we randomly selected 70% of pairs of shoes within brand and size strata; the remaining 30% of the pairs of shoes were allocated to the testing group. The resulting training data set included 42 pairs of Nike shoes (15 and 27 pairs in sizes 8.5 and 10.5, respectively) and 15 pairs of Adidas shoes (seven pairs in size 8 and eight pairs in size 10). We use the term RF‐SURF to indicate that the features are extracted from SURFs.

### 3.4 R‐package

To implement MC‐COMP, we have developed an R‐package called shoeprintr that can be used to compare shoe outsoles. The package is available on Github, in https://github.com/CSAFE-ISU/shoeprintr. The primary function in shoeprintr is *boosted*_*clique* that calculates similarity features given two sets of coordinate points after rotation and translation, based on a set of estimated parameters. The function has an option to adjust the number of points by *subsampling* points in *Q*. Subsampling is carried out in a stratified manner. First, we overlay a grid on the entire outsole to create disjoint sections of size inversely proportional to the subsampling rate we have chosen. We then select one or a few points at random from each small section. In this way, we guarantee that the subsample will cover the entire outsole. We are exploring other subsampling approaches where the size of the sections depends on the local density of pixels. To improve computational efficiency, shoeprintr is fully parallelized. This is particularly useful in the calculation of the MC used for alignment. An example of how to use the R‐package shoeprintr can be found at 22.

## 4 RESULTS

We first discuss the results we obtained when comparing pairs of good quality impressions. We consider only the more challenging case, where shoes in a pair share brand, model, size and side (left or right shoe) in both the mated and the nonmated (NM) comparisons. Differences between two shoe impressions are due to individual characteristics or randomly acquired characteristics (RACs), which can be difficult to detect by visual means, unless they are clearly marked. Mated comparisons are constructed by comparing two replicate scans of the same shoe. NM comparisons consist in comparisons between two impressions from two shoes sharing class characteristics, worn by two different participants for about 6 months.

Richetelli et al. 27 reviewed several of the methods for matching outsole impressions proposed in the literature. We implement the three best performing approaches on our dataset, to see how they compare to the method we propose. Two of the methods reviewed in Richetelli et al. 27 rely on the calculation of a phase‐only correlation coefficient (POC) and the third is based on a Fourier‐Mellin transformation correlation coefficient (FMTC). POC is not rotation invariant, so we estimate the rotation angle needed to align two impressions using a built‐in registration function called *imregtform* in Matlab for *Q* and *K*. We call this approach POC‐R. FMTC is a rotation invariant version of POC that is obtained by first by transforming images on the log polar axis system. In these data, FMTC does not exhibit good discrimination ability, so we do not discuss it further.

We computed the value of the three features and of the RF score using 681 pairs of mated (M) images and 570 NM pairs of images created from the shoes that were included in the training group. In addition to the RF, we implemented other learning algorithms and compared results, both in terms of accuracy and computational efficiency. The other algorithms included in the comparison were support vector machines, neural networks, and Bayesian additive regression trees (BART). In our application, the tree‐based algorithms outperformed the rest, at least in terms of accuracy. Predictive accuracy was essentially the same when comparing BART and RF, so both algorithms were equally well‐suited for this particular application. We focused on RF because the algorithm is efficient and results are easier to interpret.

**Figure 4:**
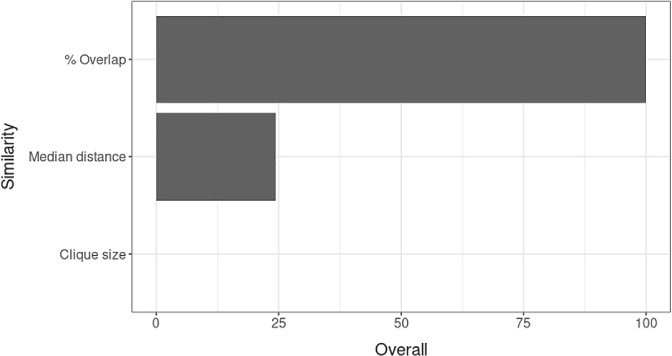
Variable importance from the trained random forest on three similarity features (clique size, % overlap and median distance)

One good feature of the RF is that it outputs an estimate of importance of each feature in the classifier. Figure 4 shows the ranking of the three features by their importance. In this particular application, the variable % overlap is clearly the most important similarity feature and appears to drive the classifier's predictive performance.

To test whether the RF can effectively determine whether a pair of images has a common or a different source, we classified 288 pairs of M and 204 pairs of NM of impressions in the testing data set. We compute the proportion of erroneous decisions and use it as a criterion to quantify the performance of the method. We also compute the precision and recall of the algorithm.

**Figure 5:**
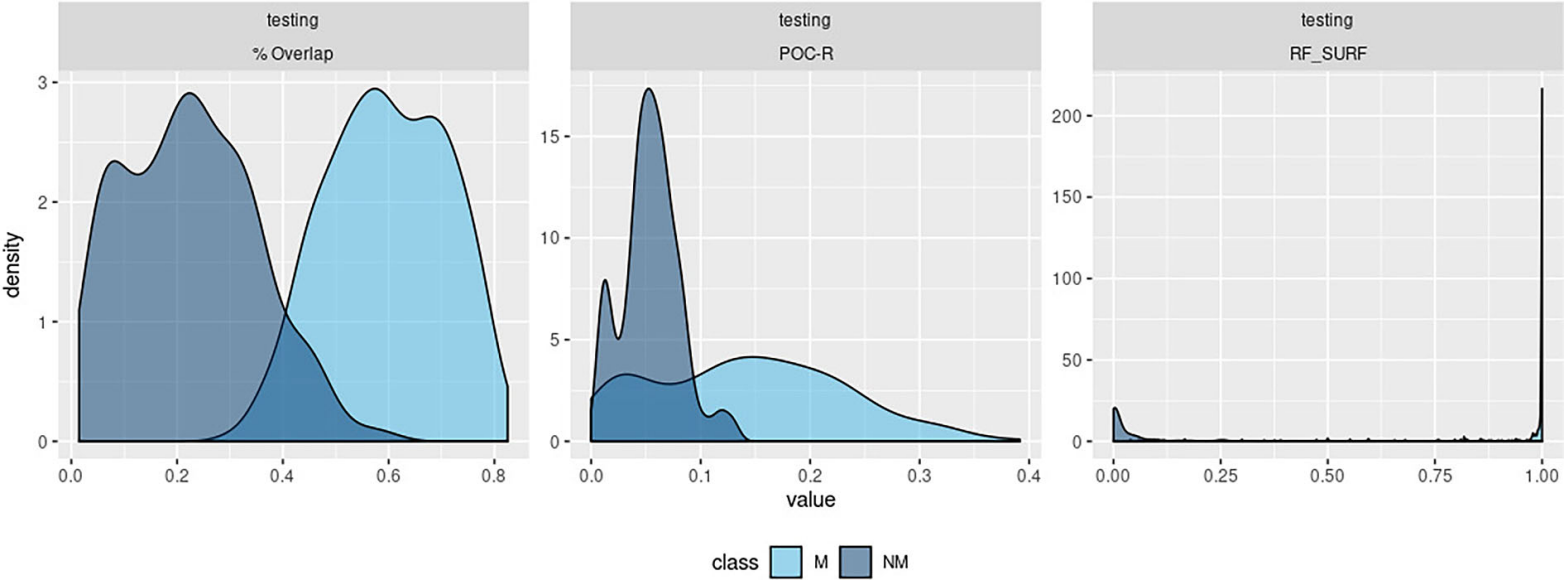
Observed densities of three similarity scores, by class in testing set

Figure 5 shows the observed distributions of three classifiers: % overlap, POC‐R, RF‐SURF computed using only the pairs of images in the testing set. Although all pairs of images include shoes that share brand and model, results show reasonably good separation by classes by the classifiers that consist of the % overlap or the POC‐R, and very good separation by the RF classifier. This suggests that when properly combined, the features extracted from the SURFs are better at discriminating M and NM pairs of images than the methods based on phase‐only correlations discussed in Richetelli et al. 27. In Figure 5, the density plot in the right most is the predicted scores in test set, which shows a very good separation between two classes.

**Figure 6:**
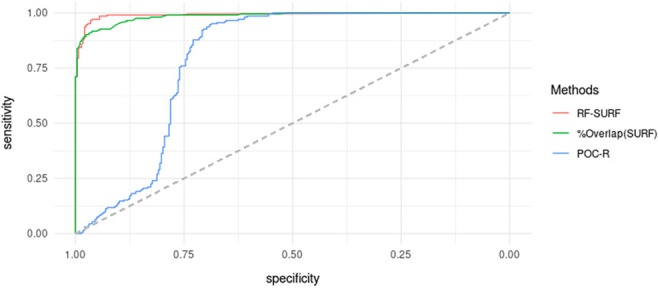
Receiver operating characteristic curves of three classifiers applied to the images in the testing data set

The ROC (receiver operating characteristic) curves shown in Figure 6 help visualize the difference in performance of the algorithms on the test images. The *x*‐axis shows the false positive rate and the *y*‐axis shows the true positive rate, so the ROC curve illustrates the trade‐offs between sensitivity (true positive rate) and specificity (one minus false positive rate) of the algorithms for a range of choices of the classification threshold. An ideal algorithm would yield 100% sensitivity and 100% specificity. Figure 6 confirms that the RF score appears to have a good classifying performance, but the simple classifier that just uses the proportion of overlapping SURFs also performs well. Table 1 shows additional diagnostics associated with the ROC curves in Figure 6. The first column in Table 1 is the estimated AUC (area under the ROC curve). The larger the AUC, the better the classifier. We also calculated the optimal threshold (OT), which we use as the cut‐off to classify pairs of images into same and different source. The OT is defined as the cut‐off that minimizes the sum of FPR and FNR (false positive and false negative rates, respectively), and is shown in the third column of Table 1. In the fourth and fifth columns, sensitivity and specificity values using the optimal threshold are reported.

**Table 1:** Area under the curve (AUC), optimal threshold (OT) to produce the lowest sum of false positive rate and false negative rate from receiver operating characteristic curves in Figure 6, sensitivity and specificity calculated using OT

Methods	AUC	OT	Sensitivity	Specificity
RF‐SURF	0.9910	0.4910	0.9706	0.9618
% Overlap (SURF)	0.9855	0.4052	0.9167	0.9618
POC‐R	0.7947	0.0958	0.9510	0.6875

Abbreviations: POC, phase‐only correlation coefficient; RF, random forest; SURF, speeded‐up robust feature.

## 5 ROBUSTNESS TO IMAGE DEGRADATION

### 5.1 Degraded images

Shoe outsole impressions found at crime scenes are often degraded in some way. They can be smudged, or partially obscured, with background effects and other noise. Therefore, any comparison algorithm that is useful in practice, must be reasonably robust to degradation of the *Q* impression (the questioned shoe outsole impression). To test whether the algorithms we discuss here can withstand degradation of *Q*, we carried out a small, preliminary experiment.

**Figure 7:**
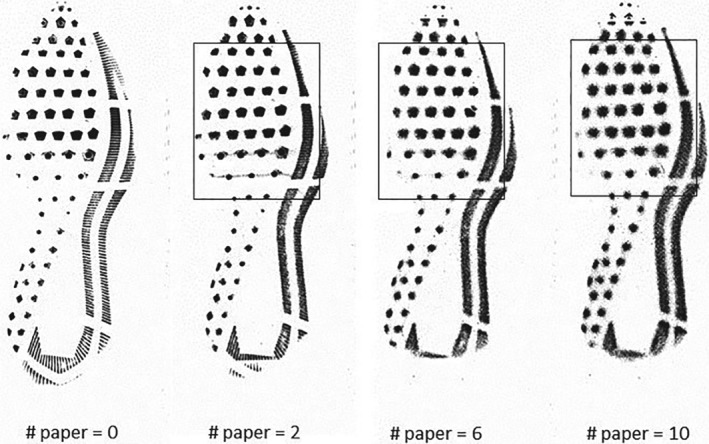
Degraded impressions from the same shoe of Nike Winflow 4. For *Q*, we used the partial impression shown in the rectangles in the three right‐most impressions. For *K*, we used a good quality image

Still using the set of Nike shoes with the same class characteristics, for each pair in the data set, we obtained a degraded *Q* image by interposing sheets of paper between the surface of the EverOS 2D scanner and the bottom of the shoe. We created a sequence of images from each shoe, each time adding more sheets of paper between the shoe outsole and the surface of the scanner. We started with two sheets of paper and increased the number by two until we reached 10 sheets of paper below the shoe. Figure 7 shows a sequence of impressions of the same shoe with increased levels of blurring. The left‐most impression in Figure 7 corresponds to the normal scan without any added noise or degradation. As more sheets of paper were placed on top of the scanner, the images became more and more smudged. In addition to blurring the images, we also deleted the portions of the *Q* images outside of the rectangular sections shown in the three right‐most impressions in Figure 7.

### 5.2 Performance on degraded images

In this section, we present the results we obtained when comparing the *K* images to the partially observed and degraded *Q* images that were obtained when 10 sheets of paper were interposed between the surface of the EverOS 2D scanner and the outsole of the shoe.

We randomly selected 24 pairs of Nike shoes (48 shoes in total) from the database, from which we constructed M and NM pairs of images using a good quality image for *K* and a partially observed and degraded image for *Q* (like the right most image in Figure 7). As before, we extracted 500 strong SURFs from each of the images. To train the random forest, we randomly selected 75% of the shoes and constructed 108 M and 108 NM comparisons. The rest of the shoes were used to make 36 M and 36 NM pairs of images to test the performance of the classifiers.

**Figure 8:**
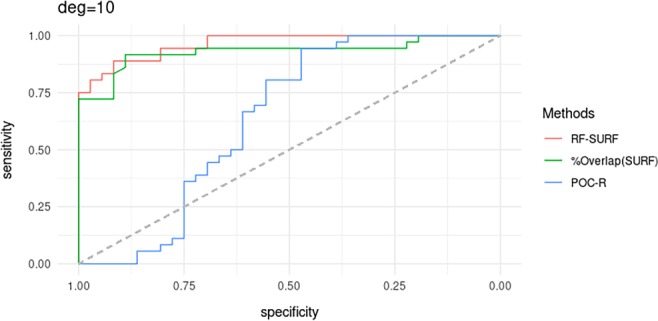
Receiver operating characteristic curves for three classifiers applied to pairs of images in the test data set where one of them is partially observed and blurry

Figure 8 shows the ROC curves that are used to compare the classification performance of three classifiers: RF‐SURF, % overlap on SURF, and POC‐R. When *Q* is degraded and partially observed, the simple classifier that consists in using the proportion of overlapping SURF pixels as the score, seems to be robust to this particular form of degradation and image fragmentation. Even when *Q* is very blurry as a consequence of the 10 sheets of paper placed between the shoe and the scanner, the single‐feature classifier exhibited high sensitivity and high specificity. The RF‐SURF classifier also performed well, but only after the RF was retrained on pairs of images where one of the images was blurred in the same manner.

The POC‐R classifier's accuracy was, however, just slightly better than what might result from flipping a coin. When *Q* is degraded and partially observed, finding an adequate rotation angle for overlaying *Q* on top of *K* was not easy, due to lack of corresponding points in *Q* for *K*. This resulted in low correlation values, which in turn led to high false negative predictions. Table 2 summarizes results. From the table, the estimated AUC is 0.96 for RF‐SURF and is 0.93 for the simple % overlap on SURF classifier.

**Table 2:** Area under the curve (AUC), optimal threshold (OT) to produce the lowest sum of false positive rate and false negative rate from receiver operating characteristic curves in Figure 8, sensitivity and specificity calculated using OT

Methods	AUC	OT	Sensitivity	Specificity
RF‐SURF	0.9645	0.5580	0.8889	0.9167
% Overlap (SURF)	0.9302	0.3839	0.9167	0.8889
POC‐R	0.6404	0.0197	0.9444	0.4722

Abbreviations: POC, phase‐only correlation coefficient; RF, random forest; SURF, speeded‐up robust feature.

## 6 DISCUSSION

Shoe outsole impressions are ubiquitous in crime scenes, yet are rarely presented as evidence in criminal proceedings. This is in part due to the fact that forensic scientists do not have reliable and objective approaches to quantify the degree of similarity between two impressions and are limited to offering their subjective opinion. Efforts to develop objective, reliable, reproducible, repeatable methods to help determine whether the suspect's shoe might have been the source of the print at the crime scene are ongoing and several approaches have been proposed in the literature. Most of the methods have been tested on datasets that do not reflect real case work, where examiners typically must compare impressions left by shoes that share class characteristics, and where the questioned print may be partially observed and subject to degradation.

Some of the recent literature 10, 26 focuses on the comparison of RACs. Because RACs arise as a consequence of wear and tear, they are believed to be unique to each shoe. Therefore, researchers are interested on the estimation of the probability of observing a specific pair of RACs on two outsole images that may be due to chance alone. Here, we consider the entire outsole and propose an approach to compare two impressions, that relies on robust and efficient features (SURFs) and on an algorithm that uses the geometric arrangement of SURFs to align both impressions. Unless RACs are faint, we would expect them to be included as part of the robust features, and therefore, as part of the classifier. The algorithm we propose is not meant to replace the examiner; rather, it is meant to serve as a quantitative measure of the similarity between two outsole impressions and to complement the examiner's expert opinion.

The algorithm we propose can be applied more broadly, in problems where the goal is to quantify the similarity between 2D images. In addition to forensic applications, images similar to the ones we have analyzed arise in biomedical, environmental, and biological applications. We are currently exploring extensions of the method to compare 3D images, which would increase the domains of application even further. In the specific case of forensic pattern matching, the alignment of features on images using the MC approach can be applied to other evidence types, including finger prints, tire treads, biometrics and other. The R‐package shoeprintr that we developed for this application, and continue to improve, has a function called *boosted*_*clique*, which implements a highly optimized algorithm to compute the MC using parallel computing. This function carries out calculations efficiently and enables application of the graph‐based alignment method in many other problems.

Another direction for extension of the method consists in increasing the number and variety of “points of interest” on which to train the algorithm; many more features can be extracted using methods such as KAZE 8, ORB 28, and SIFT 12 in addition to SURF. In this paper, we have focused on SURF to represent images, but the other approaches to extract different types of image features mentioned above can also be implemented. We fitted the RF using KAZE features, ORB features, or a combination of KAZE, ORB, and SURF features, and compared classification accuracy. In general, the RF based on KAZE or SURF features exhibited similar classification performance, both when implemented on pairs of good quality images or on pairs where one image was degraded. ORB on the other hand selects the corner pixels as features, so its performance on degraded images is poor.

We use an experimental set of impressions obtained from Nike and Adidas shoes of a limited set of sizes and that had about the same degree of wear and tear. When constructing pairs of images for comparison, we paired Nike with Nike and Adidas with Adidas, so that every pair included two impressions with the same class characteristics and a similar degree of use. While we focused on high quality images to develop our method, we created a set of partial and degraded images to explore its robustness and its usefulness in real case work.

The experimental set of images allowed us to construct comparison pairs for which we knew ground truth; the pairs of images were mated, if they corresponded to replicate impressions of the same shoe, or NM if they were obtained from different shoes. The final goal was to develop a classification algorithm that can correctly tell whether two images were mates or not. To construct such a classifier, we measured various attributes using the aligned pairs of images, and then explored whether any of those attributes could effectively discriminate M and NM pairs of images. While all attributes carried information about the source of two images, none of them could sufficiently tell mates and nonmates apart, so we combined attributes into a single score using a random forest (RF). The random forest produces a score between 0 and 1 computed as the empirical probability that the images are mates. The higher the score, the higher the probability that the images are mates.

Results are promising; on hundreds of pairs of good quality images, the RF classifier outperformed every algorithm with which we compared it. The out‐of‐bag error was a low 2%, even though comparisons included only images of shoes sharing class attributes and even though the pairs in the testing set were not used to train the algorithm. Most of the false positive results occurred with pairs of images obtained from shoes with less wear. When the latent print was degraded and only partially observed, the RF trained on good quality images suffered, but interestingly, one of the attributes we measured—the proportion of overlapping SURFs in aligned images—exhibited robustness to degradation and the fact that the *Q* was partially observed. We note that when the RF was retrained using both good quality and degraded images, its performance on a test set that included degraded images was greatly improved, as is shown in Table 2.

Before any of these automated approaches can be used in real case work, much research remains to be done. First, the algorithm we propose must undergo extensive testing and validating, using a wide arrange of outsole patterns, with varying degrees of wear and tear, and that are subject to different types of noise and degradation. In this work, we could not consider all possible types of image degradation, so we focused on increasingly smudging the *Q* image using a single tool, sheets of papers interposed between the shoe outsole and the surface of the scanner. We also deleted about 50% of *Q* in the comparisons, to mimic what may be observed at a crime scene. We fully recognize that these limited tests are just initial steps toward proposing an algorithm that is useful in a wide range of real world situations.

Second, even if we can confidently declare that two images exhibit high degree of similarity, that in no way implies that they have a common source. So the existence of an effective classifier does not address the question of the *probative value of the evidence*. We also need to show that a high degree of similarity is observed only when two impressions have a common source. To do this, we need to carefully think about the construction of large databases of outsole pattern images for which we know ground truth and that are representative of the outsole patterns that are observed in the wild. The probative value of evidence is associated with the likelihood ratio statistic, computed as the probability of observing the evidence under two competing propositions: the latent at the crime scene was made by the suspect's shoe versus the latent was made by someone else's shoe. The latter proposition must be clearly defined, and determines which images from the reference database are included in the comparison. We do not address these important issues in this article, but note that these are not easy questions and require a lot more research and collaboration with practitioners.
